# Clinicopathological Features and Prognosis of Resected Pancreatic Ductal Adenocarcinoma Patients with Claudin-18 Overexpression

**DOI:** 10.3390/jcm12165394

**Published:** 2023-08-19

**Authors:** Sejun Park, Kabsoo Shin, In-Ho Kim, Taeho Hong, Younghoon Kim, Jahee Suh, Myungah Lee

**Affiliations:** 1 Division of Medical Oncology, Department of Internal Medicine, The Catholic University of Korea, Seoul St. Mary’s Hospital, Seoul 06591, Republic of Korea; psj6936@naver.com (S.P.); agx002@naver.com (K.S.); ihkmd@catholic.ac.kr (I.-H.K.); 2 Department of General Surgery, The Catholic University of Korea, Seoul St. Mary’s Hospital, Seoul 06591, Republic of Korea; gshth@catholic.ac.kr; 3 Department of Pathology, The Catholic University of Korea, Seoul St. Mary’s Hospital, Seoul 06591, Republic of Korea; sellar1@snu.ac.kr; 4 Department of Pathology, National Medical Center, Seoul 03080, Republic of Korea; ceciliapath@nmc.or.kr

**Keywords:** pancreatic cancer, claudins, zolbetuximab, prognosis

## Abstract

Claudin-18.2 (CLDN18.2) is specifically expressed in pancreatic precancerous lesions and pancreatic ductal adenocarcinoma (PDAC). We assessed the clinical characteristics of patients with CLDN18.2-overexpressing pancreatic cancer to identify patients who might benefit from CLDN18-targeted treatment. A total of 130 patients with surgically resected PDAC were investigated for the immunohistochemical expression of claudin-18 (CLDN18). The CLDN18 staining intensities (0–3+) and relative proportion of positive tumor cells were analyzed by two independent raters. Tumors positive for CLDN18 expression were defined as ≥80% of tumor cells with 2+ or 3+ staining intensity in a CLDN18 immunohistochemical assay. Positive CLDN18 expression was present in 41/130 (31.5%) surgically resected PDACs and the relative proportion of positive tumor cells and the staining intensity were directly correlated (*p* < 0.001). Positive CLDN18 expression was significantly associated with well-differentiated tumors (*p* < 0.001) and less regional node involvement (*p* = 0.045). The positive CLDN18-expressing group showed no statistical difference in median overall survival (17.4 months vs. 20.6 months, *p* = 0.770) compared to the negative CLDN18-expressing group. Distant nodal metastasis was more frequent in the positive CLDN18-expressing group (*p* = 0.011). CLDN18 is frequently expressed in PDAC, and high CLDN18-expressing PDACs showed some different clinicopathologic characteristics. High CLDN18 expression was not associated with prognosis in patients with surgically resected PDAC.

## 1. Introduction

Pancreatic ductal adenocarcinoma (PDAC) is a major cause of cancer-related death worldwide, with most patients presenting with either unresectable or metastatic disease at the time of diagnosis [1,2]. Despite the increasing incidence and advances in treatment, the survival rate of PDAC remains low [3]. To date, only surgery remains the curative treatment modality, and the 5-year survival rate is less than 5% when curative resection is not possible [4]. Combination chemotherapy is a mainstay treatment for patients with unresectable or metastatic PDAC. The development of combination regimens, including gemcitabine with albumin-bound paclitaxel (nab-paclitaxel) and FOLFIRINOX (a combination of oxaliplatin, irinotecan, folinic acid, and fluorouracil), has improved the survival outcomes of patients with metastatic PDAC [5,6].

In the context of targeted therapy for pancreatic cancer, erlotinib, which specifically inhibits epidermal growth factor receptor 1, has been approved for the treatment of PDAC supported by clinical findings that demonstrate a modest extension in survival [7]. In the phase III POLO trial, olaparib (poly adenosine diphosphate–ribose polymerase inhibitor) was found to be an effective maintenance therapy in patients with pancreatic cancer and germline BRCA mutations [8]. In addition, larotrectinib and entrectinib, both potent and highly selective small-molecule inhibitors of all three tropomyosin receptor kinase (TRK) proteins, are feasible for patients with pancreatic cancer harboring TRK fusions [9,10]. However, these targeted agents could only be used for a small percentage of patients with the above genetic alterations. Thus, studies exploring new targeted agents are urgently required.

Claudins are a family of transmembrane tight junction proteins comprising at least 27 members [11]. These proteins predominantly reside in the apical region of the cellular membrane, where they form a paracellular barrier. This barrier regulates the passage of specific ions, including sodium (Na^+^) and chloride (Cl^-^), between adjacent cells, maintaining cellular homeostasis. They are also associated with the transduction of cell signaling pathways and the regulation of proliferation and differentiation [12,13]. Various claudins are expressed in different normal tissues and can be modified during carcinogenesis. For instance, claudin-1 is typically downregulated in breast and colon cancers [14,15]. Conversely, some claudin proteins, such as claudin-3 and claudin-4, are often upregulated in certain malignancies, including ovarian, breast, and pancreatic cancers [16,17,18]. Claudin-18 (CLDN18) is a member of the claudin family that is specifically expressed in stomach and lung tissues. Among the two CLDN18 variants produced by alternative splicing, claudin-18.2 (CLDN18.2) is specifically expressed in the gastric mucosa, while claudin-18.1 is expressed in lung tissue [19]. CLDN18.2 is aberrantly expressed not only in pancreatic cystic tumors (e.g., mucinous cystic tumors and intraductal papillary mucinous neoplasms) but also in 60–90% of PDACs [20,21,22]. Given that CLDN18.2 is not expressed in normal pancreatic tissue but is strongly activated during carcinogenesis, it could be an attractive therapeutic target for PDAC [21].

The recently developed zolbetuximab is a selective monoclonal antibody to CLDN18.2 for the treatment of gastric cancer. Zolbetuximab binds to malignant tissues that highly express CLDN18.2 without affecting healthy tissues that do not express CLDN18.2. This unique cancer-specific feature of zolbetuximab allows for maximum anticancer effects and lower toxicity [23]. In a phase II randomized trial (FAST) for advanced gastric, gastric-esophageal junction, and esophageal cancers expressing CLD18.2, the combination of zolbetuximab with cytotoxic chemotherapy achieved longer progression-free survival and overall survival (OS) than conventional chemotherapy alone [24]. The phase II trial of patients with CLDN18.2-expressing metastatic PDAC is currently ongoing and is aimed to assess the efficacy and safety of zolbetuximab in combination with gemcitabine and nab-paclitaxel as first-line treatment (ClinicalTrials.gov NCT 03816163).

This study aimed to investigate the clinicopathological features of patients with CLDN18.2-expressing PDAC and to evaluate the usefulness of CLND18.2 expression as a potential prognostic biomarker. Toward this goal, CLDN18.2 expression was analyzed immunohistochemically in PDAC patients.

## 2. Materials and Methods

### 2.1. Patients

This study evaluated 130 PDAC patients who underwent curative intent surgical resection between January 2010 and December 2018 at the Catholic University of Korea, Seoul St. Mary’s Hospital. Patients with available electronic medical records were eligible according to the following criteria: (1) histologically confirmed PDAC; (2) pathological stage I–IV disease according to the American Joint Committee of Cancer Staging, 8th edition [25]; and (3) recurrence and survival could be confirmed at the time of data collection.

### 2.2. Immunohistochemistry

Formalin-fixed and paraffin-embedded primary tumor samples were obtained and cut into 4 μm-thick sections. The rabbit monoclonal antibody anti-claudin 18 (34H14L15; 1 × 1000; Invitrogen, Carlsbad, CA, USA) was used to detect the expression and localization of CLDN18. Immunohistochemistry was performed according to established protocols. Briefly, human PDAC tissue sections were deparaffinized and rehydrated. The sections were then incubated with 3.0% H_2_O_2_ in methanol for 10 min to inhibit endogenous peroxidase activity. Antigen retrieval was performed by microwaving for 15 min. After blocking with normal goat serum for 30 min, the tissue sections were incubated with the primary antibody against claudin 18 in a humidified chamber at 4 °C overnight. Biotinylated goat anti-rabbit IgG (Vector Laboratories, Burlington, ON, Canada) was used at a 1:250 dilution as the secondary antibody. The slides were then incubated with peroxidase solution (Vectastain Elite ABC Kit, Vector Laboratories, Burlington, ON, Canada) and diaminobenzidine substrate (Vector Laboratories, Burlington, ON, Canada). Finally, the sections were counterstained with hematoxylin.

### 2.3. Histological Assessment

**Figure 1:**
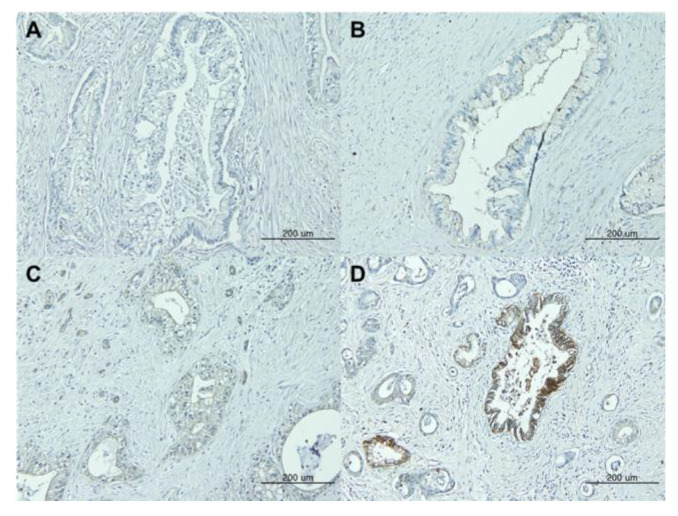
Immunohistochemical staining of claudin 18 in pancreatic ductal adenocarcinoma. Staining intensity was subclassified as (**A**) negative (0), (**B**) weak (+1), (**C**) moderate (+2), or (**D**) strong (+3).

CLDN18 expression was assessed semi-quantitatively based on the intensity of membrane staining and the percentage of tumor cells expressing CLDN18. Staining intensity was scored by two pathologists as follows: 0, +1 (weak intensity), +2 (moderate intensity), or +3 (strong intensity) (Figure 1). Positive CLDN18 expression was defined as membranous staining visible in ≥80% of the tumor cells with moderate to strong staining intensity (+2 or +3). The expression of CLDN18 was evaluated based solely on its expression in the PDAC component, excluding its expression in precancerous lesions like pancreatic intraepithelial neoplasia (PanIN). Disagreements in the assessments were resolved through a discussion to reach a consensus.

Histologic grading was determined using the criteria endorsed by the World Health Organization, referencing the Kloeppel grading scheme [26]. This method emphasizes the assessment of key features such as glandular differentiation, mucin production, mitotic activity, and nuclear pleomorphism. Notably, these features can exhibit variations within the same tumor, and the most severe grade observed is consistently reported.

### 2.4. Ethics

All methods were performed in accordance with Korean regulations and the Declaration of Helsinki. This study was approved by the Institutional Review Board (IRB) of The Catholic University of Korea, Seoul St. Mary’s Hospital (approval ID: KC21SISI0074), with a waiver of informed consent due to the retrospective nature of the analysis.

### 2.5. Statistical Analysis

Descriptive statistics were reported as proportions and medians with ranges. The association between CLDN18 expression and clinicopathological features was evaluated using Chi-squared and Fisher’s exact tests and displayed using cross-tables. Associations between staining intensity and the proportion of CLDN18-positive tumor cells were determined using an unpaired *t*-test. Recurrence-free survival (RFS) was defined as the time from surgery until the date of disease recurrence, any-cause death, or the date of last follow-up. OS was estimated from the date of surgery to the time of the last follow-up or death. Survival curves were generated using the Kaplan–Meier method and analyzed using a two-tailed log-rank test. Cox proportional hazards regression models were used to identify the effects of clinical factors on recurrence and survival. Hazard ratios (HR) and 95% confidence intervals (CIs) were estimated for each factor. All statistical analyses were performed using SPSS for Windows (version 24.0; IBM SPSS Inc., Armonk, NY, USA) and GraphPad Prism version 8.0 (GraphPad Software Inc., San Diego, CA, USA). All tests were two-sided, and *p*-values < 0.05 were considered statistically significant.

## 3. Results

### 3.1. Demographic and Clinicopathological Patient Characteristics

The demographic and clinicopathological features of the 130 patients are shown in Table 1. The median patient age was 68 years (range, 36–86 years), and the male-to-female ratio was 1.06. Approximately three-quarters (74.6%) of the patients had pancreatic head cancer, and the majority (76.2%) had moderate (grade 2) or poorly (grade 3) differentiated cancer. With respect to tumor, node, and metastasis (TNM) stage, 33 patients (25.4%) had stage I disease (IA = 5, IB = 28); 65 patients (50.0%) stage II (IIA = 7, IIB = 58); 25 patients (19.2%) stage III; and 7 patients (5.4%) stage IV. Serum carbohydrate antigen 19-9 (CA 19-9) levels were elevated in 76 patients (58.5%) at the time of diagnosis.

### 3.2. CLND18 Expression in PDAC

**Figure 2:**
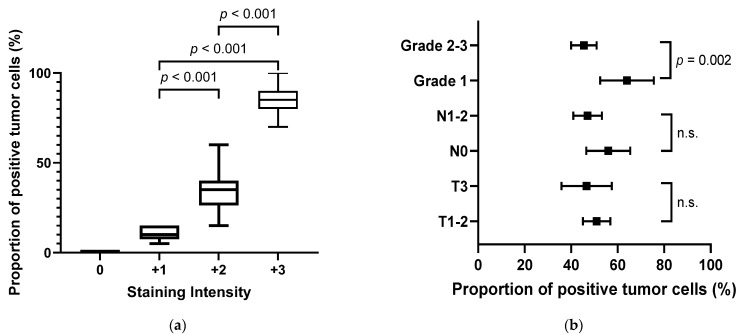
Claudin-18 (CLDN18)-expressing pancreatic cancer samples by staining intensity and proportion of positive tumor cells. (**a**) Significant correlation between relative proportion of CLDN18-positive tumor cells and staining intensity. (**b**) Association between histopathological factors and the fraction of CLDN18-positive tumor cells (bars indicate the 95% CI, n.s. indicates *p* > 0.05). T1–2, primary tumor category 1–2; T3, primary tumor category 3; N0, no regional lymph node metastasis; N1–2, metastasis in one or more regional lymph nodes; grade 1, well-differentiated; grade 2–3, moderately or poorly differentiated.

When comparing normal and reactive ducts, CLDN18 was expressed, at least focally, in 125/130 (96.2%) evaluable samples. Overall, 116 (89.2%) patients showed CLDN18 expression with a staining intensity ≥+2, and a considerable portion of patients (36.9%, n = 48) showed a strong staining intensity (+3). Interestingly, the staining intensity and the proportion of positive tumor cells were significantly correlated. Comparative analysis revealed that increased staining intensities consistently corresponded with a greater proportion of tumor cells expressing positive CLDN18. Statistically significant distinctions were observed across the comparisons: +1 vs. +2 (*p* < 0.001), +1 vs. +3 (*p* < 0.001), and +2 vs. +3 (*p* < 0.001), as illustrated in Figure 2a. In addition, although tumor and node stages were not associated with the proportion of positive tumor cells, tumor differentiation was significantly correlated with the proportion of CLDN18-expressing tumor cells (grade 1 vs. grades 2–3, *p* = 0.002, Figure 2b).

### 3.3. CLDN18 Expression and Clinicopathological Features

Overall, 41 patients (31.5%) showed positive CLDN18 expression (Table 1). Positive CLDN18 expression was more common in well-differentiated carcinomas, whereas focal or negative CLDN18 expression was more common in higher-grade carcinomas. In total, 18 (58.1%) patients with grade 1 tumor differentiation showed positive CLDN18 expression, whereas only 23 (23.2%) patients with grade 2–3 tumor differentiation showed positive CLDN18 expression (*p* < 0.001). Positive CLDN18 expression was associated with lower node stage (N0 vs. N1–2), with a higher prevalence of positive expression among the N0 stage (*p* = 0.045), but not with the tumor stage. A more advanced TNM stage was correlated with a low prevalence of positive CLDN18 expression (stage I vs. stage II–IV, *p* = 0.015). Positive CLDN18 expression was also associated with microscopic vascular invasion, with a higher prevalence of positive expression in patients without vascular invasion (*p* = 0.031). Meanwhile, positive CLDN18 expression was not correlated with age, sex, tumor location, lymphatic and perineural invasion, and preoperative CA 19-9 level. The proportion of patients who received adjuvant chemotherapy did not show a statistically significant difference between the two groups divided by CLDN18 expression.

### 3.4. CLDN18 Expression and Survival Outcomes

**Figure 3:**
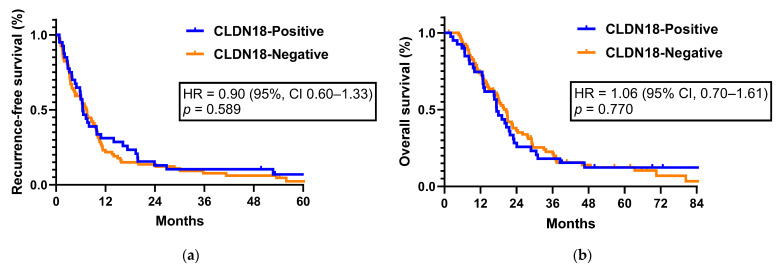
(**a**) Recurrence-free survival and (**b**) overall survival of patients with stage I-III surgically treated pancreatic cancer according to claudin-18 expression.

Among the 130 patients, 123 patients with stage I–III pancreatic cancer were included in the recurrence and survival analysis. Stage IV patients who already had distant metastatic lesions at the time of surgery were excluded from the analysis. Within a median follow-up of 17.6 months, 109 patients (88.6%) experienced recurrence and 103 patients (83.7%) died. Positive CLDN18 expression was not associated with prolonged or shortened RFS. The median RFS was not significantly different between the positive and negative CLDN18 expression groups (6.5 months (95% CI, 4.9–8.0) vs. 7.5 months (95% CI, 6.1–8.9) (HR = 0.90; 95% CI, 0.60–1.33; *p* = 0.589, Figure 3a, Table 2). In the subgroup analysis evaluating the association between CLDN18 expression and RFS stratified by each TNM stage, we did not observe a statistically significant correlation between CLDN18 expression and RFS (Figure S1A–C). Additionally, in the subgroup that received adjuvant chemotherapy, no statistically significant difference was observed in RFS based on CLDN18 expression (*p* = 0.250, Figure S2A). Univariate analysis showed that histologic grade, adjuvant chemotherapy administration, cancer stage, vascular and perineural invasion, and lymph node ratio (LNR) status were risk factors for pancreatic cancer recurrence. However, only higher histologic grade (grade 2–3 vs. grade 1, HR = 2.08; 95% CI, 1.30–3.33; *p* = 0.002), adjuvant chemotherapy (HR = 0.29; 95% CI, 0.18–0.46; *p* < 0.001), and perineural invasion (HR = 2.56; 95% CI, 1.32–4.95; *p* = 0.005) were associated with RFS after multivariate analysis (Table 2).

Positive CLDN18 expression was not associated with survival outcomes. The median OS was 17.4 months (95% CI, 14.3–20.4) in the CLDN18-positive group and 20.6 months (95% CI, 17.5–23.6) in the CLDN18-negative group (HR = 1.06; 95% CI, 0.70–1.61; *p* = 0.770, Figure 3b, Table 3). In the survival outcomes analysis stratified by TNM stage, there was no observed association between CLDN18 expression and OS (Figure S1D–F). Among patients who received adjuvant chemotherapy, OS was not significantly different between the two groups (*p* = 0.437, Figure S2B). The results of the univariate and multivariate analyses for OS are shown in Table 3, with subgroups according to clinicopathological parameters. In the multivariate analysis, higher histologic grade (grade 2–3 vs. grade 1, HR = 1.75; 95% CI, 1.07–2.84; *p* = 0.025) and vascular invasion (HR = 1.78; 95% CI, 1.15–2.77; *p* = 0.010) were significantly associated with worse OS outcomes. In addition, adjuvant chemotherapy was associated with longer OS (HR = 0.62; 95% CI, 0.40–0.98; *p* = 0.040) (Table 3).

### 3.5. CLDN18 Expression and Patterns of Recurrence or Metastasis

Recurrence was confirmed in 109/123 patients (88.6%), and seven patients had metastatic lesions at the time of surgery. The patterns of recurrence and metastatic sites are presented in Table 4. Local recurrence occurred in 26/116 patients (22.4%); distant-only recurrence in 63 patients (54.3%); and both local and distant recurrence in 27 patients (23.3%). CLDN18 expression was not associated with recurrence patterns. With respect to the number of metastatic sites and metastatic burden, a similar distribution was observed between the negative and positive CLDN18 expression groups. Interestingly, the positive CLDN18 expression group had more frequent distant nodal metastases than did the negative CLDN18 expression group (*p* = 0.011). No significant differences were observed in the other sites of metastasis (liver, lung, and peritoneum) (Table 4).

**Figure 4:**
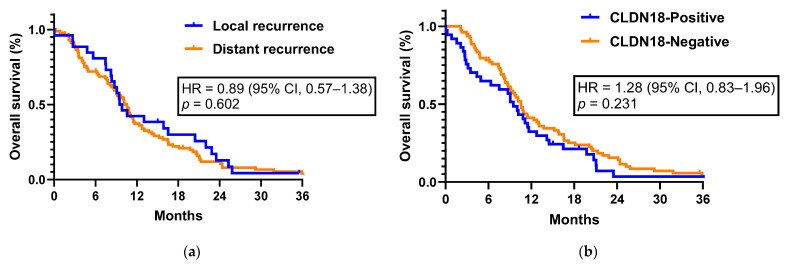
Kaplan–Meier curves showing survival from time of recurrence. (**a**) Group stratified by local versus distant disease. (**b**) Group stratified by claudin-18 expression.

The median post-recurrence survival was 9.48 months (95% CI, 7.59–11.36) for patients with local recurrence and 10.53 months (95% CI, 8.99–12.6) for those with distant recurrence (HR = 0.89; 95% CI, 0.57–1.38; *p* = 0.602, Figure 4a). The median survival from the time of recurrence to the time of any-cause death was 9.48 months (95% CI, 7.76–11.20) in the CLDN18-positive group and 10.56 months (95% CI, 9.39–11.73) in the CLDN18-negative group (HR = 1.28; 95% CI, 0.83–1.96; *p* = 0.231, Figure 4b).

## 4. Discussion

Advanced pancreatic cancer has a dismal prognosis, and no effective targeted agents have been developed for pancreatic cancer. CLDN18.2 is known to be specifically expressed in PDAC, and several clinical trials using therapeutic agents against CLDN18.2 have been conducted [27,28].

CLDN18 is frequently overexpressed in infiltrating PDAC and is overexpressed in PanIN, known as a precancerous lesion. Therefore, CLDN18 may be an early-stage marker of pancreatic carcinogenesis [29]. Previous studies have reported that CLDN18 is expressed in 60–80% of PDAC cases [21,22,29]. Our results are consistent with previous findings, with most patients (89.2%) having ≥2+ intensity of CLDN18 expression, and positive CLDN18 expression (defined as staining intensity ≥2+ and ≥80% of neoplastic cells) was observed in 41 (31.5%) of 130 patients. Additionally, CLDN18 staining intensity and the fraction of stained tumor cells were significantly correlated, consistent with previous findings [21].

We found a significant association between CLDN18 expression and tumor differentiation, with well-differentiated tumors having a higher prevalence of CLDN18-positive cases. Histologic grading is determined based on glandular differentiation, mitosis, mucin production, and nuclear pleomorphism [25]. The heterogeneity of these features within the same tumor is common; therefore, the highest grade is reported. Despite the possibility of statistical errors, a significant association was observed between tumor grade and CLDN18 expression. Additionally, in moderately or poorly differentiated tumors, the focal well-differentiated portion showed CLDN18 overexpression. In contrast, as the grade of differentiation deteriorated, CLDN18 expression decreased. This association is supported by several previous reports demonstrating that CLDN18 is overexpressed in well-differentiated pancreatic cancer and its precursors [20,21,22,29,30]. These results suggest that CLDN18 may be a marker of the early carcinogenetic process of PDAC.

Correlation analysis revealed that the proportion of tumors with positive CLDN18 expression was significantly higher in lymph-node-negative tumors, in contrast to previously reported data [21]. However, some prior studies have reported that no association was observed between node stage and CLDN18 expression, and thus, the association remains controversial [22]. We also observed significantly higher CLDN18 expression in early TNM stage and low LNR-status tumors, possibly because CLDN18 positivity was correlated with node-negative tumors. These findings could be explained by increased features of tumor stemness when CLDN18 expression was downregulated [30]. A previous study suggested that downregulated CLDN18 expression in gastric cancer cells correlates with increased cellular proliferation and epithelial-to-mesenchymal transition, both of which are associated with invasion and metastasis. These findings underscore the potential tumor-suppressive function of CLDN18 [31].

We hypothesized that CLDN18 expression might be associated with prognosis based on the association between CLDN18 expression and the aforementioned prognostic factors. However, there was no relationship between survival and CLDN18 expression. Only histologic grading and adjuvant chemotherapy were correlated with RFS and OS outcomes. There was no significant difference in the rate of adjuvant chemotherapy according to CLDN18 expression (25/40 patients (62.5%) in the CLDN18-positive group and 59/83 patients (71.1%) in the CLDN-negative group. In a prior study examining 160 surgically treated pancreatic cancer patients, survival outcomes were associated with CLDN18 expression. Specifically, patients demonstrating strong and diffuse CLDN18 expression showed improved survival outcomes (HR = 0.52, 95% CI, 0.32–0.84) relative to those manifesting weak or absent claudin 18 expression [22]. However, this study did not clearly describe the stage, and only 43% of the registered participants received adjuvant treatment, which poses limitations to the interpretation of the results. Contrarily, another investigation involving 111 pancreatic cancer patients found no prognostic correlation tied to CLDN18 expression patterns [30]. Thus, the prognostic value of CLDN18 expression remains unclear and requires further investigation.

The current study found no significant difference in the pattern of recurrence according to the CLDN18 expression. We initially hypothesized that patients with positive CLDN18 expression would have a less aggressive tumor biology than those with negative CLDN18 expression. However, there was no difference observed in the pattern of recurrence, number of metastatic sites, or metastatic burden according to the CLDN18 expression. With respect to organotropism, distant nodal metastasis was observed at a significantly higher prevalence in the CLDN18-positive group than in the CLDN18-negative group. Additionally, lung metastasis tended to be more frequent in the CLDN18-negative group, although the difference was not significant. Metastatic organotropism varies among host organs with different abilities to colonize cancer cells [32]. Further studies are needed to identify the host factors associated with distant nodal metastasis in patients with CLDN18-expressing PDAC.

Similar to recurrence, survival outcomes also did not differ according to CLDN18 expression. However, these results may not be sufficient to evaluate the prognostic impact of CLDN18 expression in palliative settings because we did not assess CLDN18 expression in recurrent or metastatic samples. A previous study investigated CLDN18 expression in matched samples of primary and metastatic lesions, and the majority of patients had comparable CLDN18 expression in both lesions [21]. However, the study had a small sample size, and thus, further studies are needed to validate previous results.

This study systematically investigated CLDN18 expression in surgically treated patients with PDAC. To the best of our knowledge, this study is the first to evaluate the clinicopathological features and patterns of recurrence in patients with CLDN18-positive pancreatic cancer. Considering CLDN18 as a potential therapeutic target for pancreatic cancer, our results may be beneficial in selecting subjects for CLDN18-targeted treatment in the future. However, this study also had some limitations. First, we employed an antibody targeting CLDN18 instead of one specifically binding to CLDN18.2. Additionally, the criteria for determining CLDN18 positivity were delineated based on our own criteria. Clinically meaningful criteria and detection methods need to be established according to the results of ongoing clinical trials of zolbetuximab, and accompanying diagnostic modalities and cutoff values also need to be determined. Second, treatment differed among patients, and this could not be controlled owing to the retrospective nature of the study. Third, the relatively small sample size limited the interpretation of the subgroup analysis according to CLDN18 expression. Further studies are warranted to identify patients who may benefit from CLDN18-targeted treatments.

In conclusion, CLDN18 overexpression is correlated with several clinicopathological features, but not with prognosis in pancreatic cancer. Patients with a high proportion of well-differentiated histologic pancreatic cancer could be evaluated for CLND18 expression for possible targeted therapy in the future.

## Figures and Tables

**Table 1:** Clinicodemographic patient characteristics by CLDN18 expression.

Variables	Total (*n* = 130)	CLDN18 Negative (*n* = 89)	CLDN18 Positive (*n* = 41)	*p* Value
Age, Median (Range)	68 (36–86)	68 (36–81)	63 (39–86)	0.219
Gender, *n* (%)				0.421
Male	67 (51.5)	48 (71.6)	19 (28.4)	
Female	63 (48.5)	41 (65.1)	22 (34.9)	
Tumor location, *n* (%)				0.542
Head	97 (74.6)	65 (67.0)	32 (33.0)	
Body/Tail	33 (25.4)	24 (72.7)	9 (27.3)	
Histologic grading, *n* (%)				<0.001
Grade 1	31 (23.8)	13 (41.9)	18 (58.1)	
Grade 2/3	99 (76.2)	76 (76.8)	23 (23.2)	
Tumor stage, *n* (%)				0.542
T1–2	97 (74.6)	65 (67.0)	32 (33.0)	
T3	33 (25.4)	24 (72.7)	9 (27.3)	
Node stage, *n* (%)				0.045
N0	41 (31.5)	23 (56.1)	18 (43.9)	
N1–2	89 (68.5)	66 (74.2)	23 (25.8)	
TNM stage *, *n* (%)				0.015
Stage I	33 (25.4)	17 (51.5)	16 (48.5)	
Stage II/III/IV	97 (74.6)	72 (74.2)	25 (25.8)	
Lymphatic invasion, *n* (%)				0.902
No	39 (30.0)	27 (69.2)	12 (30.8)	
Yes	91 (70.0)	62 (68.1)	29 (31.9)	
Vascular invasion, *n* (%)				0.031
No	55 (42.3)	32 (58.2)	23 (41.8)	
Yes	75 (57.7)	57 (76.0)	18 (24.0)	
Perineural invasion, *n* (%)				0.979
No	16 (12.3)	11 (68.8)	5 (31.2)	
Yes	114 (87.7)	78 (68.4)	36 (31.6)	
Lymph node ratio, *n* (%)				0.038
≤0.1	71 (54.6)	43 (60.6)	28 (39.4)	
>0.1	59 (45.4)	46 (78.0)	13 (22.0)	
Received adjuvant CTx, *n* (%) **				0.338
No	39 (31.7)	24 (61.5)	15 (38.5)	
Yes	84 (68.3)	59 (70.2)	25 (29.8)	
Regimen of adjuvant CTx, *n* (%) †				0.709
Gemcitabine	19 (22.6)	14 (73.7)	5 (26.3)	
5-FU/Leucovorin	65 (77.4)	45 (69.2)	20 (30.8)	
Preoperative CA19-9 level, *n* (%)				0.737
Within normal (<40 U/mL)	39 (30.0)	26 (66.7)	13 (33.3)	
Above normal (≥40 U/mL)	76 (58.5)	53 (69.7)	23 (30.3)	
Missing data	15 (11.5)			

CLDN18, claudin-18; TNM, tumor, node, metastasis; CA 19-9, carbohydrate antigen 19-9; CTx, chemotherapy; 5-FU, fluorouracil. * According to the American Joint Committee on Cancer, 8th edition. ** In the population excluding stage IV patients (*n* = 123). ^†^ In the population who received adjuvant chemotherapy (*n* = 84).

**Table 2:** Univariate and multivariate analyses of the association between clinicopathologic features and CLDN18 expression for recurrence-free survival.

	RFS			
Variables	Univariate Analysis		Multivariate Analysis	
	HR (95% CI)	*p* Value	HR (95% CI)	*p* Value
Age ≥ 65 (vs. <65 year)	0.93 (0.64–1.36)	0.705		
**Histologic grade 2–3 (vs. grade 1)**	**2.06 (1.30–3.27)**	**0.002**	**2.08 (1.30–3.33)**	**0.002**
**Received adjuvant chemotherapy (vs. none)**	**0.48 (0.31–0.73)**	**0.001**	**0.29 (0.18–0.46)**	**<0.001**
Stage II (vs. Stage I)	1.68 (1.06–2.67)	0.027	1.26 (0.74–2.13)	0.391
Stage III (vs. Stage I)	2.46 (1.39–4.35)	0.002	1.56 (0.71–3.44)	0.272
Lymphatic invasion (vs. none)	1.49 (0.98–2.27)	0.064		
Vascular invasion (vs. none)	1.62 (1.10–2.40)	0.015	1.40 (0.93–2.11)	0.109
**Perineural invasion (vs. none)**	**1.97 (1.10–3.54)**	**0.022**	**2.56 (1.32–4.95)**	**0.005**
LNR > 0.1 (vs. ≤0.1)	1.70 (1.16–2.50)	0.007	1.30 (0.75–2.24)	0.352
CLDN18-positive (vs. negative)	0.90 (0.60–1.33)	0.589		

CLDN18, claudin-18; RFS, recurrence-free survival; HR, hazard ratio; LNR, lymph node ratio. Statistically significant variables are in bold font.

**Table 3:** Univariate and multivariate analyses of the association between clinicopathologic findings and CLDN18 expression for overall survival.

	OS			
Variables	Univariate Analysis		Multivariate Analysis	
	HR (95% CI)	*p* Value	HR (95% CI)	*p* Value
Age ≥ 65 (vs. <65 year)	1.01 (0.68–1.49)	0.968		
**Histologic grade 2–3 (vs. grade 1)**	**1.91 (1.19–3.07)**	**0.007**	**1.75 (1.07–2.84)**	**0.025**
**Received adjuvant chemotherapy (vs. none)**	**0.71 (0.46–1.09)**	**0.118**	**0.62 (0.40–0.98)**	**0.040**
Stage II (vs. Stage I)	1.39 (0.86–2.23)	0.179	1.09 (0.66–1.78)	0.743
Stage III (vs. Stage I)	2.23 (1.25–3.97)	0.007	1.75 (0.94–3.27)	0.077
Lymphatic invasion (vs. none)	1.58 (1.03–2.42)	0.036	0.98 (0.59–1.61)	0.923
**Vascular invasion (vs. none)**	**1.89 (1.26–2.82)**	**0.002**	**1.78 (1.15–2.77)**	**0.010**
Perineural invasion (vs. none)	2.21 (1.17–4.15)	0.014	1.96 (0.97–3.84)	0.051
LNR > 0.1 (vs. ≤0.1)	1.42 (0.96–2.10)	0.076		
CLDN18-positive (vs. negative)	1.06 (0.70–1.61)	0.770		

CLDN18, claudin-18; OS, overall survival; HR, hazard ratio; LNR, lymph node ratio. Statistically significant variables are in bold font.

**Table 4:** Patterns of recurrence and metastatic sites according to CLDN18 expression.

Variables	Total (*n* = 116)	CLDN18 Negative (*n* = 79)	CLDN18 Positive (*n* = 37)	*p* Value
Site of recurrence, *n* (%)				0.732
Local only	26 (22.4)	19 (24.1)	7 (18.9)	
Distant only	63 (54.3)	43 (54.4)	20 (54.1)	
Local and distant	27 (23.3)	17 (21.5)	10 (27.0)	
Number of metastatic sites, *n* (%)				0.279
Oligometastatic	71 (61.2)	51 (64.6)	20 (54.1)	
Polymetastatic	45 (38.8)	28 (35.4)	17 (45.9)	
Metastatic burden, *n* (%)				0.523
0–2	80 (69.0)	53 (67.1)	27 (73.0)	
≥3	36 (31.0)	26 (32.9)	10 (27.0)	
Liver metastasis, *n* (%)				0.506
No	74 (63.8)	52 (65.8)	22 (59.5)	
Yes	42 (36.2)	27 (34.2)	15 (40.5)	
Lung metastasis, *n* (%)				0.088
No	100 (86.2)	65 (82.3)	35 (94.6)	
Yes	16 (13.8)	14 (17.7)	2 (5.4)	
Peritoneal metastasis, *n* (%)				0.591
No	84 (72.4)	56 (70.9)	28 (75.7)	
Yes	32 (27.6)	23 (29.1)	9 (24.3)	
Distant nodal metastasis, *n* (%)				0.011
No	81 (69.8)	61 (77.2)	20 (54.1)	
Yes	35 (30.2)	18 (22.8)	17 (45.9)	

CLDN18, claudin-18.

## Data Availability

The data presented in this study are available on request from the corresponding author.

## Supplementary Materials

The following supporting information can be downloaded at: https://www.mdpi.com/article/10.3390/jcm12165394/s1, Figure S1: Recurrence-free survival of patients with pancreatic cancer according to claudin-18 expression stratified by pathologic stage: (A) stage I, (B) stage II, and (C) stage III. Overall survival stratified by the same stages is as follows: (D) stage I, (E) stage II, and (F) stage III; Figure S2: (A) Recurrence-free survival and (B) overall survival of patients with pancreatic cancer who received adjuvant chemotherapy according to claudin-18 expression.
