# Supplementary material for: Clinicopathological Features and Prognosis of Resected Pancreatic Ductal Adenocarcinoma Patients with Claudin-18 Overexpression
Source: J Clin Med. 2023 Aug 19;12(16):5394. doi: 10.3390/jcm12165394 (PMC10455540; doi:10.3390/jcm12165394)
Supplement: Supplementary file 1 [file jcm-12-05394-s001.zip › jcm-2484510-supplementary.pdf]

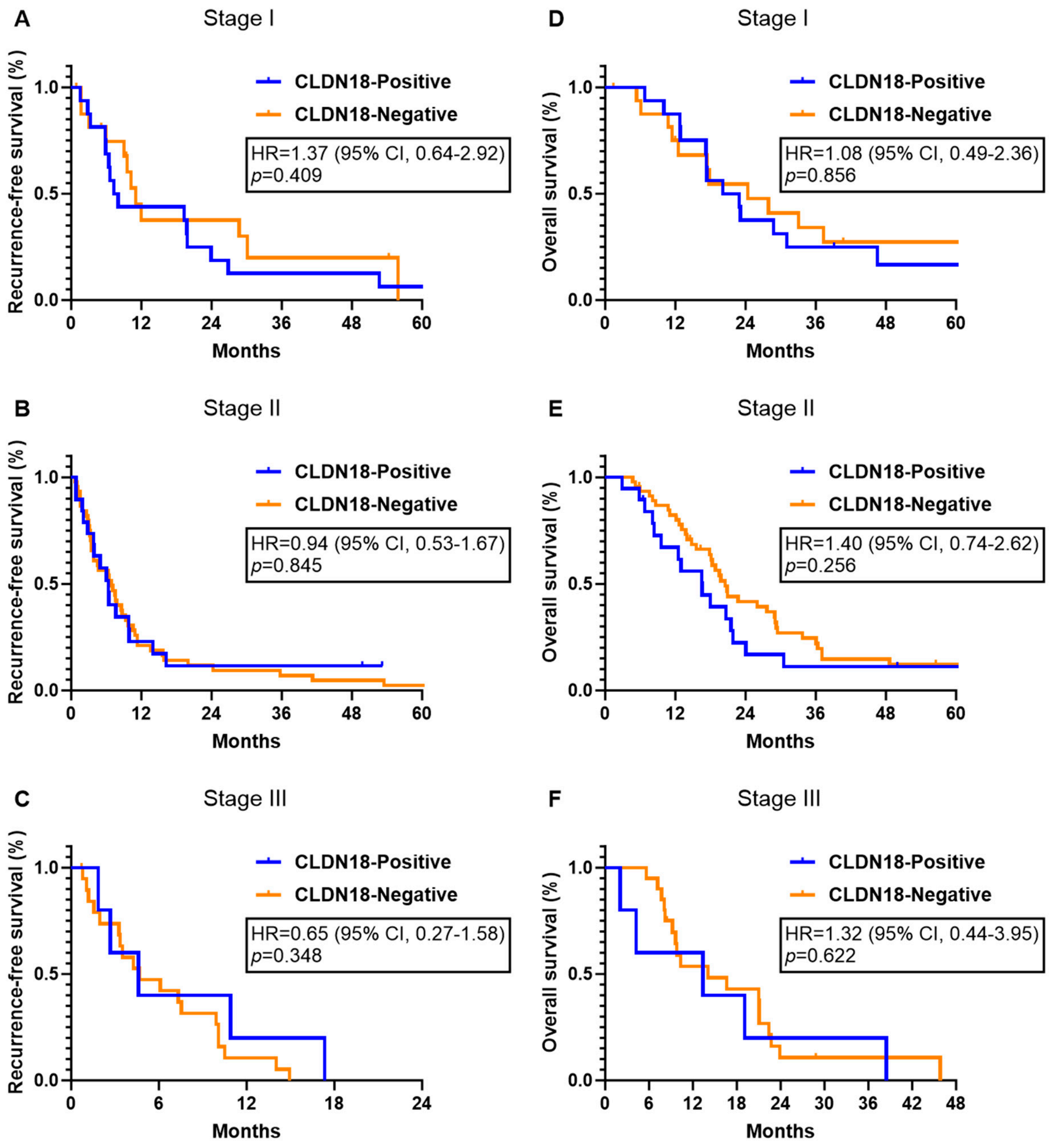

Figure S1. Recurrence-free survival of patients with pancreatic cancer according to claudin-18 expression stratified by pathologic stage: (**A**) stage I, (**B**) stage II, and (**C**) stage III. Overall survival stratified by the same stages is as follows: (**D**) stage I, (**E**) stage II, and (**F**) stage III.

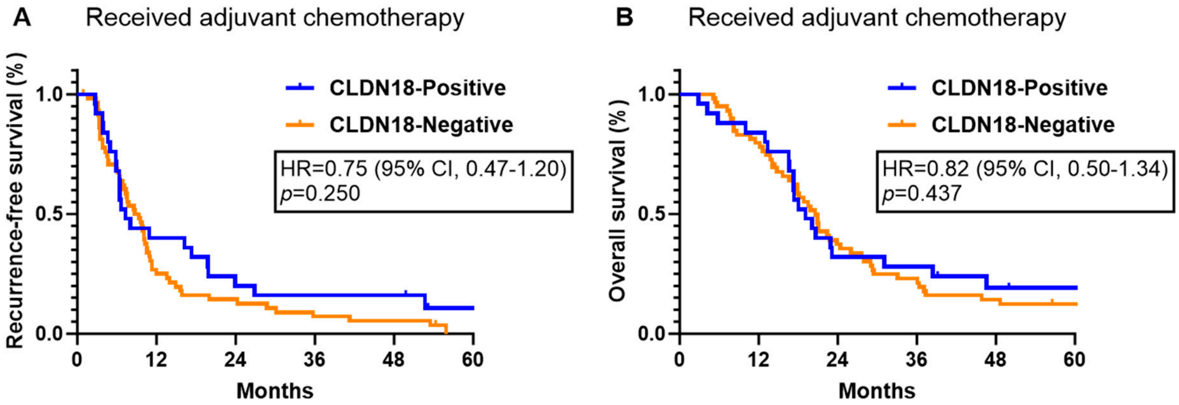

Figure S2. (**A**) Recurrence-free survival and (**B**) overall survival of patients with pancreatic cancer who received adjuvant chemotherapy according to claudin-18 expression.
